# Case report: Primary Ewing sarcoma of the ureter, an exceptional finding of unique manifestation of disease

**DOI:** 10.3389/fonc.2022.1070838

**Published:** 2023-01-05

**Authors:** Marina Valeri, Leocadia Dore, Noemi Rudini, Miriam Cieri, Grazia Maria Elefante, Alberto Saita, Alexia Bertuzzi, Piergiuseppe Colombo

**Affiliations:** ^1^ Department of Biomedical Sciences, Humanitas University, Milan, Italy; ^2^ Department of Pathology, Humanitas Research Hospital, IRCCS, Milan, Italy; ^3^ Department of Urology, Humanitas Research Hospital, IRCCS, Milan, Italy; ^4^ Department of Oncology, Humanitas Research Hospital, IRCCS, Milan, Italy

**Keywords:** Ewing sarcoma, ureter, extraskeletal ES, *EWSR1–FLI1*, rare tumor, case report

## Abstract

Ewing sarcoma (ES) is the second most common malignant bone tumor in children and has also been described in adults with highly aggressive behavior. ES belongs to the small round blue cell tumor family and presents the distinctive translocation of FET-ETS family genes (85% with *EWSR1*), generating gene fusions. Extraskeletal ES mainly occurs in soft tissues; the urogenital tract is rarely affected, and ureteral localization is an exceptional event with only 4 cases described in the literature. Here we report the first Italian case of primary ES of the ureter, a 24-year-old young man with lower back pain and a narrowed left ureteral lumen on CT scan. ES of the urogenital tract is an almost unique condition with a nonspecific clinical presentation and a challenging diagnosis for pathologists. We encourage awareness of these exceptional events in the differential diagnosis of ureteral lesions in young patients.

## 1 Introduction

Ewing’s sarcoma (ES) is a malignant neoplasm with highly aggressive behavior (1). It is the second most common malignant bone tumor in children, but less frequent cases have been described in older patients, often affecting extraskeletal sites (2).

ES is characterized and defined by the presence of non-random chromosomal translocations—detectable by fluorescence *in situ* hybridization (FISH)—producing fusion genes that involve one member of the FET family of genes and a member of the E26 transformation-specific (ETS) family (*EWSR1–FLI1* in 85%–90% of cases), encoding aberrant transcription factors (3). Histologically, classical ES consists of a proliferation of uniformly small round cells with inconspicuous nucleoli and scant clear to pale cytoplasm, with a sheet-like growth pattern (4). Tumor cells usually show strong and diffuse expression of CD99 and NKX2.2 (5). The current management of ES includes neoadjuvant chemotherapy followed by surgical resection and/or radiotherapy and adjuvant chemotherapy. Systemic treatment is usually composed of multiagent chemotherapy cycles of vincristine, doxorubicin, and cyclophosphamide (VDC) alternating with cycles of ifosfamide and etoposide (IE) (6).

ES usually affects bone, but it can also localize in extraskeletal sites, most commonly in soft tissues (12% of cases) (1). Urogenital tract involvement is a rare event, most frequently arising in the kidney, followed by the bladder and the prostate (7–11). As for the ureter, a primary ES is an exceptional finding: to our knowledge, there are no more than four cases described in the literature (12–15).

Here we report the fifth case of primary ureteral ES and the first in Italy as a unique manifestation of the disease.

## 2 Case description

**Figure 1:**
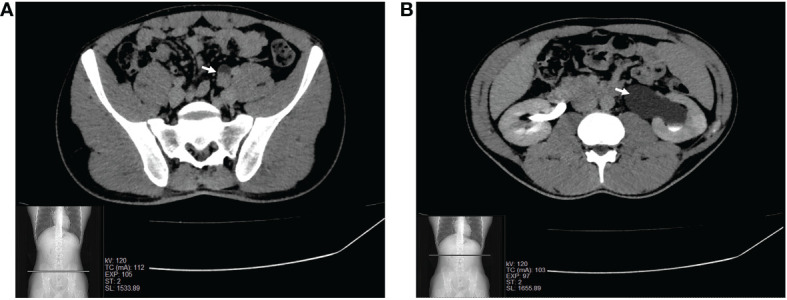
CT scan of the urological tract scan showing the narrowed lumen of the left pelvic ureter **(A)** and ipsilateral hydronephrosis **(B)**.

A 24-year-old male presented to our institution with a 6-month history of left lower back pain. Five years before, he underwent surgery for varicocele and had a history of prostatitis. No relevant family medical history was documented. Abdominal US and CT scans of the urological tract with contrast medium were performed with the finding of a narrowed left ureteral lumen and ipsilateral hydronephrosis (**Figure 1**). Cytology was negative for malignancy, and subsequent diagnostic ureteroscopy revealed a polypoid fibrotic lesion, but a biopsy was not feasible. To resolve the obstruction and understand the nature of the lesion, the patient underwent a left distal segmental ureterectomy with intraoperative evaluation of the specimen.

**Figure 2:**
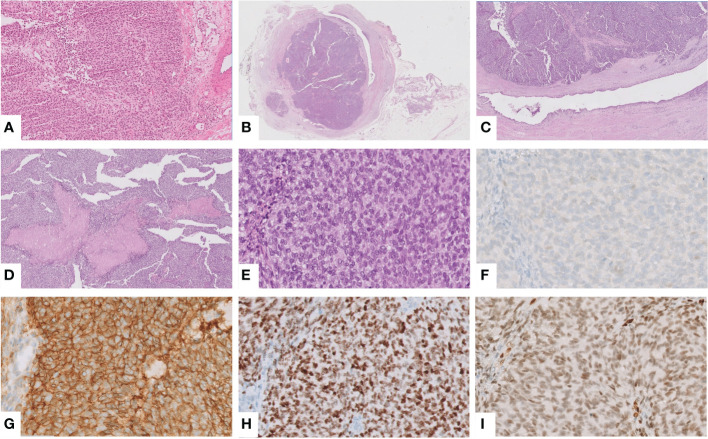
Intraoperative frozen section showing a solid proliferation of round to ovoid epithelioid cells, with atypical nuclei and eosinophilic cytoplasm, suggesting a urothelial carcinoma (hematoxylin and eosin (H&E), ×10) **(A)**. An obstructing mass was found in the ureteral wall (H&E, ×0.25) **(B)**, which corresponded to a solid growth with spared overlining urothelium (H&E, ×2) **(C)** and foci of necrosis (H&E, ×5) **(D)**. Notably, the tumor was composed of round to spindle, monotonous, small cells with the peculiar intracytoplasmic glycogen (H&E, ×40) **(E)**. Immunohistochemically, the tumor was negative for GATA3 **(F)** but showed the characteristic positivity for CD99 **(G)**, NKX2.2 **(H)** and FLI1, the suspected and most common partner of translocation, confirmed at NGS analysis **(I)**.

Frozen sections showed a proliferation of round to ovoid epithelioid cells, arranged in solid sheets, with nuclear atypia and eosinophilic cytoplasm (**Figure 2A**). These findings, together with the clinical presentation, initially suggested a urothelial carcinoma. Therefore, robot-assisted uretero-ureteral termino-terminal anastomosis and ureteral double-J stenting were performed.

**Figure 3:**
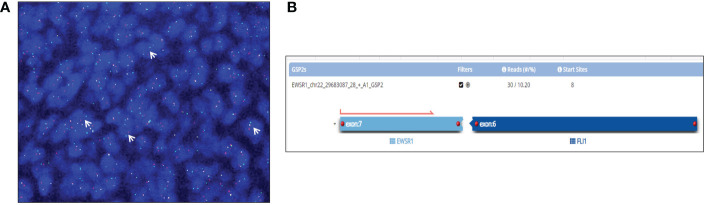
FISH with an EWSR1 Dual Color Break Apart Rearrangement Probe detected *EWSR1* translocation in 80% of tumor cells as orange and green spitting signals (white arrows) **(A)**. Pan Solid Tumor v2 NGS analysis revealed the gene fusion involving exon 7 of *EWSR1* and exon 6 of *FLI*, detected by a gene specific primer (GSP2) specific for *EWSR1* **(B)**.

Grossly, an obstructing grayish mass of 2 cm was found in the ureteral wall. Histological examination demonstrated solid growth of round to spindle cells, monotonous, small-cell malignant proliferation, and foci of necrosis. Remarkably, the overlying urothelium was spared (**Figures 2B–E**). To confirm the urothelial nature of the neoplasm, an immunohistochemical stain for GATA3 was performed, with a negative result (**Figure 2F**). These features prompted us to consider a non-urothelial tumor and to rule out small, round, blue-cell tumors. Indeed, an epithelial neoplasm was excluded by immunonegativity for panCK; tumor cells were positive for CD99, FLI1, NKX2.2, synaptophysin (only focal), and negative for myogenin, ERG, and WT1 (**Figures 2G–I**). To detect *EWSR1* translocation, FISH was performed on 4-mm-thick formalin-fixed, paraffin-embedded (FFPE) tumor sections. Vysis LSI EWSR1 (22q12) Dual Color Break Apart Rearrangement Probe (Abbott Molecular, Abbott Park, IL), a mixture of two probes that map the 5’ side of the *EWSR1* gene (labeled in SpectrumOrange) and the 3’ side of the *EWSR1* gene (labeled in SpectrumGreen), has been used. Signals were evaluated in at least 100 tumor nuclei per specimen. *EWSR1* translocation was detected in 80% of cells in the tumor area (**Figure 3A**). Eventually, primary ureteral ES was diagnosed. Subsequently, Archer Pan Solid Tumor v2 next-generation sequence analysis (NGS) was performed. Archer Data Analysis Software 6.0 confirmed *FLI-1* as the translocation partner (**Figure 3B**).

Post-surgical CT total body and PET scan did not show any distant metastases. After a definitive diagnosis and multidisciplinary tumor board, systemic multiagent adjuvant chemotherapy was administered with four cycles of vincristine (2 mg), doxorubicin (37.5 mg/m^2^), and cyclophosphamide (600 mg/m^2^) (VDC) alternating with five cycles of ifosfamide (3 g/m^2^) and etoposide (150 mg/m^2^) (IE). Subsequently, the patient underwent radicalization surgery with a partial ureterectomy and surrounding soft tissue resection, without residual tumor. After 15 months of follow-up, the patient was alive with no sign of recurrence or metastatic disease on CT total body and with programmed maintenance chemotherapy (VC×3/IE×2).

**Figure 4:**
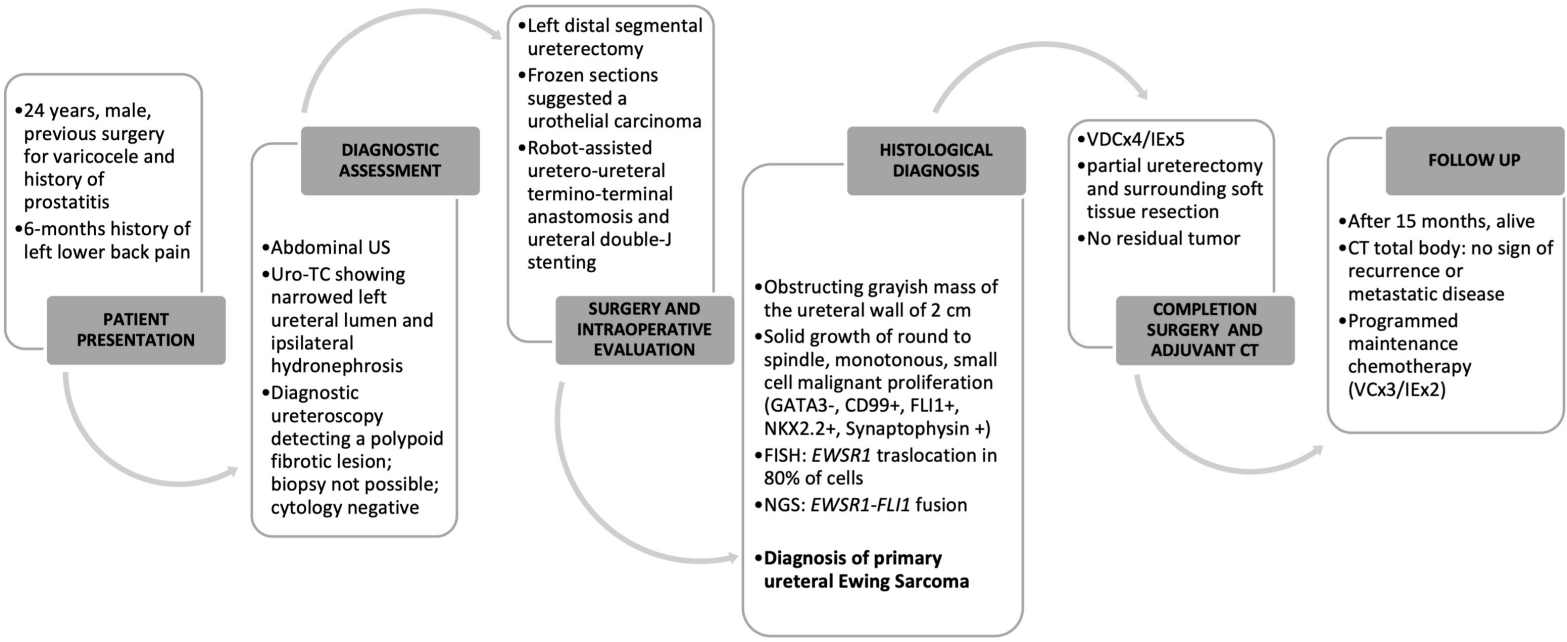
Temporal timeline.

A temporal timeline of the most relevant events is depicted in **Figure 4**.

## 3 Discussion

ES is a small round cell sarcoma characterized by FET-ETS family gene translocations (*EWSR1–FLI1* in 85%–90% of cases) (2). It is an aggressive disease usually treated with surgical excision preceded by neoadjuvant CT and followed by adjuvant CT (6).

ES usually affects the bone of children and young adults, but it can also arise in older patients and localize in extraskeletal sites, mainly in soft tissues (1). A review of the English-language literature revealed that the urogenital tract is rarely involved: the most common site is the kidney, with almost 200 cases described (7, 8), followed by the bladder (9), and the prostate (10, 11). Ureteral localization is an exceptional finding, with only four cases of primary ES of the ureter previously reported (12–15).

**Table 1** summarizes the patients’ clinical features. As for the literature cases, two patients were males, and the median age was 39.5 years. All patients had clinical symptoms as the initial presentation of the disease. Hematuria and flank pain were the most frequent symptoms (3/4), but only in two cases where they present simultaneously. Indeed, our patient presented with lower back pain without hematuria. All patients underwent surgery, and three received adjuvant systemic therapy, like in our case. All tumors showed typical features of ES. Similarly, our case presented morphological features of the classic ES and also the characteristic immunohistochemical profile with positivity for CD99, NKX2.2, FLI1, and synaptophysin (4, 5). Our Archer Pan Solid Tumor v2 NGS analysis revealed a gene fusion involving exon 7 of *EWSR1* and exon 6 of *FLI*, in keeping with the literature. Indeed, the most common breakpoints are exons 7 and 6 of *EWSR1* and exons 7 and 5 of *FLI1* (3). Interestingly, the *EWSR1–FLI1* fusion was identified only in one other patient by RT-PCR (12). Only one patient had a fatal outcome. This patient was a 45-year-old male with a deferred diagnosis of a recurrent lesion of the ureter treated with segmental ureteral resection and partial cystectomy. After 7 years, he experienced pelvic recurrence and progression to metastatic disease regardless of adjuvant chemotherapy with three courses of vincristine, adriamycin, cyclophosphamide (VAC), and IE (13).

**Table 1:** Clinical features.

Case	Reference	Age (years)	Sex	Location	Presentation	Treatment	Recurrence or Metastasis	Follow up (mo)	Status	Positive IHC staining	Molecular Test
**1**	Charny et al. (12)	17	F	Right	Flank pain, hematuria	Surgery, AD chemotherapy	NA	NA	NA	CD99	EWS–FLI1 fusion (RT-PCR)
**2**	Huang et al. (13)	45	M	Left	Hematuria	Surgery, AD chemotherapy, palliative RT	Local recurrence and lung metastasis	24	Died of disseminated metastasis	CD99, NSE, S-100, FLI1	NA
**3**	Song et al. (14)	12	M	Right	Abdominal pain	Surgery, AD chemotherapy	No	8	Alive	CD99, NSE, Vimentin	EWS translocation (FISH)
**4**	Li et al. (15)	69	F	Left	Flank pain, hematuria	Surgery	No	6	Alive	CD99, TLE1	NA
**5**	Current	24	M	Left	Lower back pain	Surgery, chemotherapy	No	15	Alive	CD99, FL1, NKX 2.2, synaptophysin (focal)	EWS translocation (FISH), EWS–FLI1 Fusion (Archer)

F, female; M, male; AD, adjuvant; RT, radiotherapy; IHC, immunohistochemistry; TLE1, Transducin-Like Enhancer of split 1; NSE, Neuron-Specific Enolase; FISH, Fluorescence *in situ* hybridization; NA, not assessed.

In a clinical scenario with a young patient presenting with non-specific symptoms and a ureteral mass, in the absence of other primitive tumors, like in our case, the differential diagnosis should comprise a localization of either a germ cell tumor or a lymphoma. Blood markers and imaging to detect abdominal lymphadenopathy might be helpful in this setting. For our patient, the CT scan excluded further abdominal lesions, while blood markers were not tested.

Although less frequent, a retroperitoneal soft tissue tumor should also be ruled out.

In the suspected case of primary urothelial carcinoma of the ureter, our patient underwent surgery without a previous diagnosis. This is because the lesion could not be biopsied during diagnostic ureteroscopy due to lumen fibrotic obstruction. Therefore, an intraoperative evaluation was performed on the partial ureterectomy specimen. Frozen sections were somehow misleading since they suggested a urothelial neoplasm. Indeed, the tumor showed a more epithelioid morphology with round to ovoid cells with eosinophilic cytoplasm, growing in solid sheets with a fair degree of atypia. The urothelial nature was also consistent with site and clinical presentation, although in a young patient. The results of the intraoperative evaluation prompted the urologist to perform an uretero-ureteral anastomosis without radicalization.

The clinical management after definitive diagnosis was challenging and required several multidisciplinary tumor boards to decide for a completion surgery and adjuvant chemotherapy. In daily practice, a preoperative diagnosis of ES is of paramount importance to the oncologist since these tumors—of any site—undergo standardized protocols with preoperative neoadjuvant chemotherapy (6). Indeed, the difficulty in obtaining adequate diagnostic material during ureteroscopy in our case had an actual impact.

Remarkably, due to the lack of a preoperative histological diagnosis, three of the four previously reported cases received chemotherapy only after surgery (12–14). In one patient, a 69-year-old woman, systemic therapy was not administered due to age and impaired cardiopulmonary function (15).

To the best of our knowledge, this is the fifth reported case of primary ureteral ES and the first in Italy. ES of the urogenital tract is an exceptional condition with nonspecific clinical presentation and a challenging diagnosis, with a possible impact on oncological management due to the common lack of preoperative histological information precluding neoadjuvant treatment protocols. We encourage awareness of these exceptional events in the differential diagnosis of ureteral lesions in young patients.

## Data availability statement

The original contributions presented in the study are included in the article/supplementary material. Further inquiries can be directed to the corresponding author.

## Ethics statement

Written informed consent was obtained from the patient for the publication of any potentially identifiable images or data included in this article.

## Author contributions

MV and PC conceived, designed, wrote, and revised the final manuscript. LD reviewed the literature, provided figures and tables, and revised the final manuscript. MC and GE reviewed the histological slides, critically revised the work, and approved the final manuscript. NR performed molecular analyses. AS performed surgery. AB followed the patient and provided clinical data. All authors contributed to the article and approved the submitted version.
